# Clinical factors increasing discrepancies of renal function assessment with MDRD and Cockcroft–Gault equations in old individuals

**DOI:** 10.1007/s41999-018-0076-9

**Published:** 2018-06-14

**Authors:** Jerzy Chudek, Aureliusz Kolonko, Aleksander J. Owczarek, Katarzyna Wieczorowska-Tobis, Katarzyna Broczek, Anna Skalska, Andrzej Więcek

**Affiliations:** 1 Pathophysiology Unit, Department of Pathophysiology, Medical Faculty in Katowice, Medical University of Silesia in Katowice, Medyków 18, 40-752 Katowice, Poland; 2 Department of Internal Medicine and Oncological Chemotherapy, Medical Faculty in Katowice, Medical University of Silesia in Katowice, Reymonta 8, 40-027 Katowice, Poland; 3 Department of Nephrology, Transplantation and Internal Medicine, Medical Faculty in Katowice, Medical University of Silesia in Katowice, Francuska 20-24, 40-027 Katowice, Poland; 4 Department of Statistics, Department of Instrumental Analysis, School of Pharmacy with the Division of Laboratory Medicine in Sosnowiec, Medical University of Silesia, Ostrogórska Str. 30, Sosnowiec, 41-200 Katowice, Poland; 5 Department of Palliative Medicine, Laboratory for Geriatrics, Poznan University of Medical Sciences, Rusa Str. 55, 61-245 Poznan, Poland; 6 Department of Geriatrics, Medical University of Warsaw, Oczki 4, 02-007 Warsaw, Poland; 7 Department of Internal Medicine and Gerontology, Jagiellonian University Medical College, Śniadeckich 10, 31-531 Kraków, Poland

**Keywords:** Activities of daily living, Age, Drug dosage, Older patients, Gender, Glomerular filtration rate

## Abstract

**Abstract:**

In a daily clinical practice, glomerular filtration rate (GFR) is still estimated on the basis of short MDRD formula, whereas medications’ Summaries of Product Characteristics suggest that GFR used for the dosage adjustment should be estimated based on the Cockcroft–Gault (C–G) equation. The aim of the study was to compare eGFR values calculated on the basis of short and full MDRD and C–G equations in PolSenior study participants with decreased eGFR.

**Methods:**

We have assessed differences in the estimation of GFR between short and full MDRD, as well as C–G formula, all equations utilizing non-isotope-dilution mass spectrometry-calibrated measurements of serum creatinine, in the community-based population of 760 persons aged 65 years or above (mean age 82 ± 8 years) with estimated GFR < 60 ml/min/1.73 m^2^ (according to short MDRD). In addition, in our analysis, we have included the detailed characteristics of comorbidities and different aspects of mobility and functional performance.

**Results:**

The better concordance, precision, and accuracy with MDRD short formula were found for MDRD_full_ than C–G equation. In logistic regression analysis, female gender, activities in daily living (ADL) ≤ 4, and age > 80 years diminished, while visceral obesity improved accuracy (P_30_) of eGFR calculated according to C–G equation as compared to MDRD_short_. Similar analysis did not found factors influencing P_30_ for MDRD_full_ equation.

**Conclusions:**

In very old subjects, especially females, dependent patients and those with visceral obesity, estimation of GFR based on short MDRD formula should not be used interchangeably with Cockcroft–Gault equation for the medicines dose tailoring.

## Introduction

The accurate estimation of renal function in older patients remains a serious challenge. For many years, the estimation was based on the Cockcroft–Gault (C–G) formula [1], regardless of noticeable limitations [2]. To mention just a few, the C–G formula underestimates GFR in slim and overestimates GFR in obese older patients [3]. Low muscle mass (sarcopenia) and decreased daily creatinine production explains the bias in GFR estimation [4]. In addition, it should be stressed that the C–G formula has never been validated in an older population. A newer equation, the MDRD formula, was introduced in late 1990s as a result of Modification of Diet in Renal Disease study analysis [5] and has gained worldwide acceptance, especially its simplified, short version including only demographic variables and serum creatinine levels. This formula has enabled an automatic calculation of GFR in all laboratories performing basic serum creatinine measurement. Of note, more recent guidelines [6] suggest the use of creatinine-based CKD-EPI equation that requires serum creatinine measurement using specific assays with calibration traceable to the isotope-dilution mass spectrometry (IDMS). The same is true for a dedicated older Berlin Initiative Study 1 equation (BIS1) [7]. However, the availability of IDMS-calibrated assays in daily clinical practice is still limited. The rising gap between recommendations and daily clinical practice is further puzzled by requirements of pharmacotherapy [8]. According to medications’ Summaries of Product Characteristics, GFR used for the dosage adjustment should be estimated based on the C–G equation. However, laboratory platforms automatically calculate GFR, usually based on MDRD formula, and listed this value in the laboratory reports, while C–G creatinine clearance has to be calculated manually by the physician. This procedure increases the risk of inappropriate drug dosing in older patients.

The aim of this study was to assess the clinical factors, including patients’ comorbidity and functional performance measures, which may increase the incompatibility of renal function assessment with MDRD or Cockcroft–Gault equations in old individuals.

## Methods

### Study design and setting

The study protocol complied with the Declaration of Helsinki regarding ethical conduct of research involving human subjects and was approved by the Bioethics Committee of Medical University of Silesia in Katowice. All participants gave their informed consent. Serum samples used for the assessment of serum creatinine in the presented study were obtained from participants of the PolSenior study—a large, cross-sectional, multicenter, interdisciplinary, publicly funded research project, focused on older people, conducted in Poland from 2007 to 2011. The study design was described elsewhere [9]. The eGFR values were available in 1906 respondents aged 65–80 years and 1592 above the age of 80 years.

Only participants with eGFR < 60 ml/min/1.73 m^2^ (according to short MDRD) were included in the current analysis.

### Laboratory measurements

Serum and urine creatinine concentrations were assessed using Jaffe method, serum urea by kinetic UV method, and serum albumin by colorimetric method (Modular PPE, Roche Diagnostics GmbH, Mannheim, Germany).

Urinalysis was performed initially with the strip method (Combur-Test) with urinary albumin quantification using Miditron M system (Roche Diagnostics GmbH, Mannheim, Germany). In subjects in whom albuminuria was not detected with this method (< 300 mg/l), high sensitivity (< 3 mg/l) immunoturbidimetric method was used (Roche Diagnostics GmbH, Mannheim, Germany). Urine albumin-to-creatinine ratio (UACR) was calculated.

Serum C-reactive protein concentrations were assessed by an automated system (Modular PPE, Roche Diagnostics GmbH, Mannheim, Germany) with intermediate precision below 5.7%.

Serum N-terminal prohormone for brain natriuretic peptide (NT-proBNP) was assessed using the electrochemiluminescence method on Cobas E411 analyzer (Roche Diagnostics GmbH, Mannheim, Germany) with intermediate precision below 4.6%.

### Data analysis

Renal function was estimated using short MDRD, full MDRD [5], and Cockcroft–Gault [1] equations. We used eGFR values for short MDRD formula to score CKD stages as recommended by KDIGO [6].

Obesity and visceral obesity were classified according to the WHO and IDF criteria [10, 11].

Diagnosis of diabetes was established based on medical history, medication, and fasting serum glucose concentration ≥ 126 mg/dl.

Cardiovascular diseases included coronary artery disease and stroke diagnoses established on the basis of a questionnaire survey and medical history. Arterial hypertension was diagnosed based on home blood pressure measurements during two visits, if the average systolic blood pressure was at least 140 mmHg and/or average diastolic blood pressure was at least 90 mmHg, or the subject reported receiving antihypertensive medications.

Subjects with serum NT-proBNP concentration above 2000 pg/ml were classified as having heart failure [12].

Assisted mobility was assessed on the basis of self-reported data. Activities of daily living (ADL) were evaluated on the basis of 6-item Katz index and the values ≤ 4 were scored as dependence.

Relative bias of eGFR values (%) was calculated as a difference between values obtained using the analyzed formulas and short MDRD equation value, expressed as the percentage of the latter.

### Statistical analysis

Statistical analysis was performed using STATISTICA 13.1 PL (StatSoft, Cracow, Poland), StataSE 12.0 (StataCorp LP, TX, USA), and R software. Statistical significance was set at a *p* value below 0.05. All tests were two-tailed. Imputations were not done for missing data. Nominal and ordinal data were expressed as percentages, whilst interval data were expressed as mean value ± standard deviation in the case of normal distribution or as median/interquartile range in the case of data with skewed or non-normal distribution. Distribution of variables was evaluated by the Shapiro–Wilk test and homogeneity of variances was assessed by the Levene test. For comparison of eGFR values between different equations, the one-way ANOVA analysis was used with Tukey’s post hoc test. To assess the influence of gender and CKD groups to relative bias, the two-way ANOVA analysis was used with contrast analysis. To assess the relationship between eGFR values calculated according to short MDRD equation and other formulas, the Passing–Bablok regression as well as the concordance correlation coefficient were used. Bias, precision, and accuracy were measured to determine the performance of each equation. Bias was calculated as a median difference with 95% confidence interval between short MDRD equation and other two formulas. Positive/negative bias means that eGFR values calculated with any formula are overestimated/underestimated in comparison to eGFR values calculated based on short MDRD equation. Precision was assessed with as interquartile range (IQR) for the differences. Accuracy was measured as the percentage of estimates within 30% of short MDRD equation (P_30_).

To identify factors affecting the relative bias of each eGFR equation, the least angle square regression was used, which provided estimate of standardized regression coefficient *β*. Positive *β* of any significant variable, enclosed in the model, means that the relative bias increases, while the variable values increase, whereas negative *β* means that the relative bias decreases, while the variable values increase. Multivariable stepwise backward logistic regression was used to identify factors affecting accuracy. The course of the relative bias was shown with the distance-weighted least-squares regression.

## Results

### Participants

Glomerular filtration rate values were estimated in 3498 subjects (85.3% out of 4101 PolSenior study participants in whom the blood samples were obtained), 1658 females (719 aged more than 80 years), and 1840 males (873 aged more than 80 years). Of all, 760 participants (416 female and 344 male) had eGFR values below 60 ml/min/1.73 m^2^, as calculated with short MDRD formula, and were included in the present analysis (Table 1).

**Table 1:** Study group characteristics’—PolSenior participants with eGFR values below 60 ml/min/1.73 m^2^ (calculated with short MDRD formula)

	All *N* = 760	Men *N* = 344	Women *N* = 416
Age, year	82 ± 8	83 ± 8	82 ± 8
BMI, kg/m^2^	28.4 ± 5.0	27.4 ± 4.1	29.2 ± 5.5
Obesity, *n* (%)	237 (31.3)	72 (20.9)	165 (39.9)
Waist circumference, cm	99.0 ± 13.0	101.2 ± 12.0	97.2 ± 13.5
Visceral obesity, *n* (%)	643 (85.3)	265 (77.7)	378 (91.5)
Assisted mobility, *n* (%)	258 (33.9)	112 (32.6)	146 (35.1)
ADL ≤ 4, *n* (%)	89 (11.7)	37 (10.8)	52 (12.5)
Hypertension, *n* (%)	588 (77.7)	250 (73.1)	338 (81.4)
Type 2 diabetes, *n* (%)	207 (27.3)	86 (25.1)	121 (29.1)
Heart failure, *n* (%)	105 (13.9)	59 (17.3)	46 (11.1)
History of stroke, *n* (%)	87 (11.5)	39 (11.4)	48 (11.6)
hs-CRP ≥ 3 mg/L, *n* (%)	356 (46.9)	168 (49.0)	188 (45.2)
UACR ≥ 300 mg/g, *n* (%)	44 (5.8)	26 (7.6)	18 (4.3)
Kidney function estimates			
Short MDRD, ml/min/1.73 m^2^	48.6 ± 8.8	48.9 ± 8.8	48.4 ± 8.8
Full MDRD, ml/min/1.73 m^2^	42.5 ± 8.2	42.7 ± 8.2	42.4 ± 8.2
Cockcroft–Gault, ml/min	42.4 ± 13.8	42.3 ± 13.3	42.6 ± 14.2

Mean ± standard deviation

*BMI* body mass index, *ADL* activities of daily living, Katz index, *hs-CRP* high-sensitive C-reactive protein, *UACR* urine albumin-to-creatinine ratio

### Comparison of eGFR values (relative bias)

The estimated slope and intercept of the Passing–Bablok regression analysis showing the relation between eGFR calculated according to short MDRD formula and eGFR yielded by two other equations, in subjects with eGFR < 60 ml/min/1.73 m^2^ (MDRD_short_), are presented in Table 2. For both examined equations (full MDRD and C–G), the slope was significantly different from the value of 1.0. Moreover, there was a negative systematic difference in both equations. Larger difference was noted in case of C–G than in full MDRD equation.

**Table 2:** Results of the Passing–Bablok regression, bias, precision, and accuracy between eGFR calculated according to C–G and MDRD_full_ equations, and concordance correlation coefficients in subjects with eGFR < 60 ml/min/1.73 m^2^ according to short MDRD

Equation	Intercept (± 95% CI)	Slope (± 95% CI)	Residual standard deviation (± 95% CI)	Concordance coefficient *ρ*_c_ (± 95% CI)	Bias median difference	Precision IQR	Accuracy P_30_
	Systematic difference	Proportional difference	Random difference		(± 95% CI)	(± 95% CI)	(± 95% CI)
C–G	− 45.93 (− 54.32– to 38.58)	1.79 (1.65–1.97)	6.70 (− 13.13–13.13)	0.455 (0.410–0.498)	− 6.79 (− 7.61 to − 5.86)	15.19 (13.92–16.46)	70.3 (66.9–73.4)
MDRD_full_	− 3.03 (− 3.94 to − 2.17)	0.94 (0.92–0.96)	1.70 (− 3.33–3.33)	0.762 (0.741–0.782)	− 5.94 (− 6.19 to − 5.72)	3.26 (2.98–3.54)	99.9 (99.3–100.0)

*IQR* interquartile range, *C–G* Cockcroft–Gault, *MDRD* modification of diet in renal disease formula

Moreover, the concordance correlation coefficient *ρ*_c_, which evaluates the degree to which pairs of observations fall on the 45° line through the origin, and, therefore, shown a level of agreement between two methods, was calculated. The higher value and thus the better concordance were obtained for full MDRD equation. In addition, bias as well as precision and accuracy were better in full MDRD equation in comparison to C–G equation.

### Factors influencing the relative bias in GFR estimation

For participants with eGFR lower than 60 ml/min/1.73 m^2^ (MDRD_short_), there was a significant influence of gender on the relative bias for full MDRD but not for the C–G equation. For full MDRD equation, men presented slightly, but significantly higher relative bias than women (Table 3). Significant differences in relative bias between particular CKD stages (G3a–G4) occurred only in case of full MDRD equation, with the relative bias increasing across CKD stages both in men and women (Table 3).

**Table 3:** Results of two-way analysis of variance for relative bias (%) in C–G and MDRD_full_ equation, according to gender and CKD stages

Relative bias (%)	All		Men			Women			*p*		
	Men *N* = 344	Women *N* = 416	G3a *N* = 237	G3b *N* = 94	G4 *N* = 13	G3a *N* = 286	G3b *N* = 109	G4 *N* = 20	Gender	CKD stages	Interaction
C–G	− 13.6 ± 21.3	− 12 ± 24.4	− 14.0 ± 21.7	− 12.9 ± 21.6	− 12.0 ± 11.8	− 12.5 ± 23.4	− 10.7 ± 26.9	− 11.5 ± 26.0	0.643	0.733	0.978
MDRD_full_	− 12.9 ± 4.6	− 12.4 ± 4.8	− 12.4 ± 4.6	− 13.7 ± 4.3	− 17.0 ± 5.3	− 12.1 ± 4.7	− 13.2 ± 4.9	− 13.6 ± 4.0	< 0.05	< 0.001	0.199

Mean ± standard deviation

*Interaction* interaction between gender and CKD stages

To identify factors affecting the relative bias of each eGFR equation, results of the least angle square regression were used (Table 4). The coefficient values are yielded for the minimum estimation of prediction error *C*_p_. For each analyzed equation, age over 80 years was the most important factor influencing the relative bias. Moreover, dependence in daily living, the occurrence of heart failure, and UACR ≥ 300 mg/g were among the most important factors affecting full MDRD equation, while the occurrence of visceral obesity, hypertension, and diabetes were the factors affecting the relative bias for C–G equation.

**Table 4:** Results of the least angle square regression for the relative difference in eGFR values (left panel) and multivariable stepwise backward logistic regression for P30 inaccuracy (right panel), calculated according to short MDRD equation, the coefficient values for the minimum estimation of prediction error *C*_p_, accordance of eGFR < 60 between short MDRD and C–G and MDRD_full_ equation

Variable	LARS regression for eGFR differences		Logistic regression for P30 inaccuracy OR (± 95% CI)	
	C–G	MDRD_full_	C–G	MDRD_full_
Diabetes	0.0799^3^	–	–	–
Heart failure	− 0.0057^8^	− 0.0078^2^	–	–
Hypertension	0.0366^4^	0.0050^5^	–	–
History of stroke	0.0047^10^	0.0065^7^	–	–
Visceral obesity	0.1663^2^	0.0049^6^	4.51^#^ (2.81–7.22)	–
hs-CRP ≥ 3	0.0185^7^	− 0.0020^8^	–	–
Assisted mobility	− 0.0101^9^	0.0063^4^	–	–
Age > 80 years	− 0.2685^1^	− 0.0141^1^	0.21^#^ (0.14–0.33)	–
ADL ≤ 4	− 0.0383^5^	–	0.60* (0.37–0.99)	–
Female gender	− 0.0299^6^	–	0.46^#^ (0.32–0.67)	–
UACR ≥ 300 mg/g	–	− 0.0249^3^	–	–

Left panel: superscript indexes show actions along the sequence of models (1 means the strongest effect). Right panel: P30: difference to group without disease as a baseline

*Hs-CRP* high-sensitive C-reactive protein, *ADL* Independence in Activity of Daily Living index, *UACR* urine albumin-to-creatinine ratio

*p < 0.05; **p <  0.01; ^#^*p* < 0.001

Logistic regression analysis, for the accuracy of eGFR calculated according to C–G equation as compared to MDRD_short_, showed that female gender, ADL ≤ 4, and age > 80 years were factors diminishing accuracy (P_30_), while visceral obesity improved accuracy (Table 4).

**Fig. 1:**
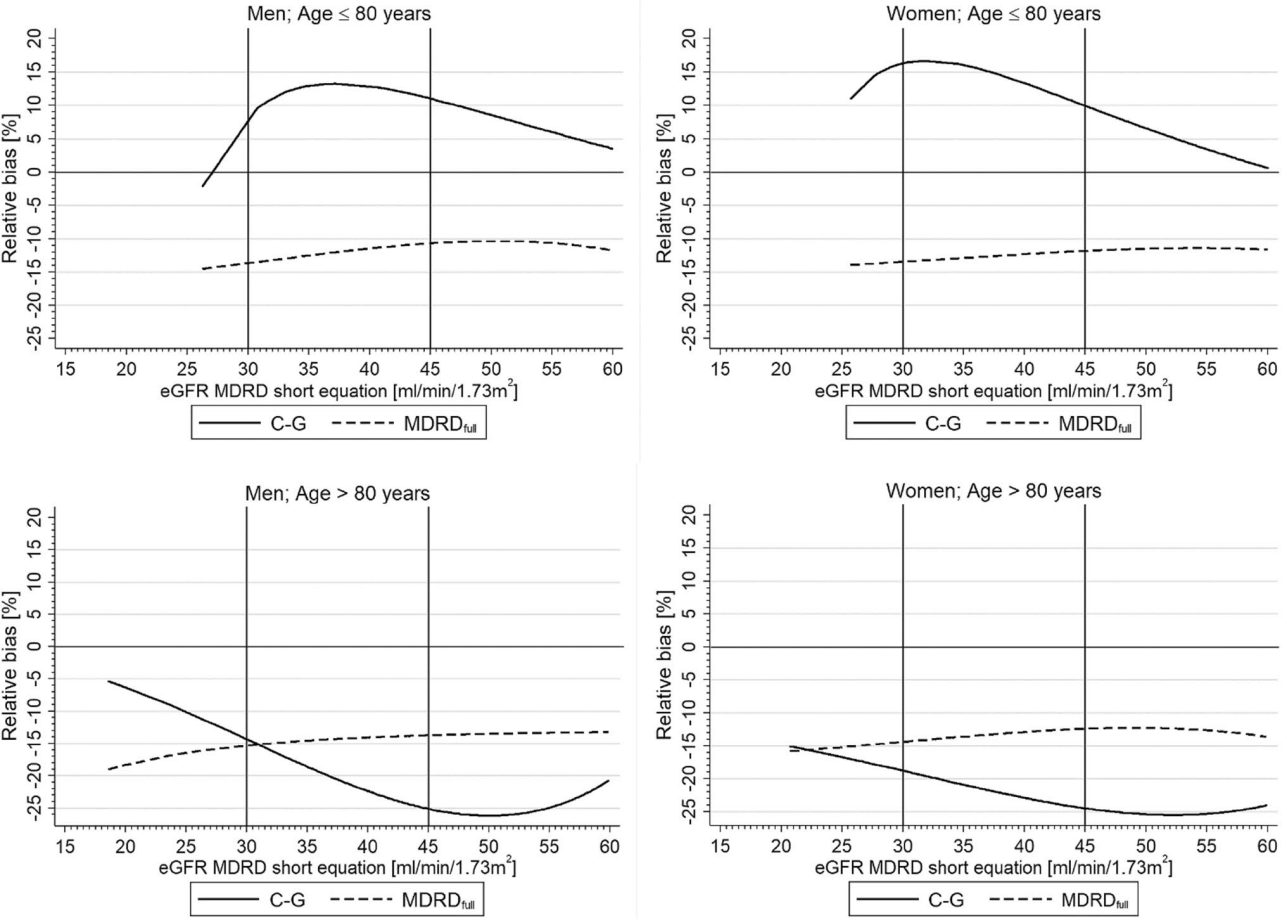
Relative bias (%) for each eGFR equation across eGFR calculated with short MDRD formula

Figure 1 presents the relative bias (%) for C–G and MDRD_full_ equation across the whole eGFR range, calculated with short MDRD equation, and shown in separate charts for men and women, and for ≤ 80 and > 80 years age groups, respectively. The relative bias of full MDRD equation remained nearly constant across eGFR values and did not differ between all four subgroups. For the C–G equation, both in men and women ≤ 80 years, the relative bias was increasing with declining of eGFR to values around 35 ml/min/1.73 m^2^ and then was diminishing, while, for older participants, the relative bias was decreasing constantly with decreasing eGFR values (from value around 50 ml/min/1.73 m^2^). Moreover, for participants ≤ 80 years, the relative bias tended to be positive, while for older ones—negative.

## Discussion

In the present study, we have analyzed the differences in the estimation of GFR between the short MDRD formula and two other equations utilizing not-IDMS-calibrated measurements of serum creatinine in the older population. As the possible clinical consequences of discrepancies between eGFR values calculated with different formulas are most relevant among patients with substantially diminished renal excretory function, we have focused on the subgroup of patients with short MDRD eGFR < 60 ml/min/1.73 m^2^. Of importance, despite of comparable mean eGFR values in the whole-study group, calculated with two different formulas, e.g., the full MDRD and the C–G, the detailed analysis revealed that there were major relative bias size as well as changes of its sign, which yield differences between gender and age subgroups. The stage of CKD was another important factor modifying the relative bias of MDRD_full_ equation, mostly in men. Moreover, comorbidity as well as the activities of daily living have further influenced the between-formula relative differences. Of note, the particular importance of all those analyzed co-factors differed widely, depending on certain eGFR formula. Neglecting of those details when calculating eGFR value in daily clinical practice may lead to the significant error in the estimation of renal excretory function in an individual patient level and, in consequence, to the inadequate diagnosis and potentially inappropriate therapy.

Our observation regarding the serious inconsistency between an acceptable MDRD and C–G average concordance with substantial differences at the individual level are in line with results of Pedone et al. [13], where only diagnosis of severe CKD (eGFR < 30 ml/min/1.73 m^2^) in older patients showed a fair agreement of two compared formulas, and the magnitude of the differences in GFR estimates was influenced by age and weight. More recently, Evans et al. have noticed an inaccuracy of five different eGFR formulas in patients with eGFR < 30 ml/min/1.73 m^2^, especially in older patients and those with diabetic nephropathy [14]. They also observed the lowest accuracy of the C–G (approximately 54%) [14]. Of note, in their study, the median bias was stable across measured GFR categories, whereas, in our study, the relative bias variability was significant regarding full MDRD equation. However, in a subgroup of patients over 65 years [14], the relative accuracy of all analyzed formulas except the C–G was inferior as compared to younger subjects.

When comparing the non-IDMS traceable eGFR formulas with the CKD-EPI equation, Bevc et al. demonstrated that the accuracy within 30% of estimated ^51^Cr-EDTA clearance values differs according to the stage of CKD [15]. Moreover, they have validated a simple cystatin C formula as a reliable marker of GFR in older persons, comparable to other formulas, including MDRD and CKD-EPI formulas. Importantly, in a recent study, Kilbride et al. have found the accuracy of short MDRD equation inferior to the CKD-EPI only in older patients with measured GFR > 60 ml/min/1.73 m^2^ [16]. Furthermore, in the very old population (mean age 85 years), the CKD-EPI and the Berlin Initiative Study (BIS) creatinine–cystatin C equations showed a better accuracy than other equations in GFR estimation [17]. The best performance of the CKD-EPI creatinine–cystatin C equation in older people was also confirmed in community-based AGES-Reykjavik Study [18]. In contrast, in the older Chinese cohort, the bias of the CKD-EPI creatinine–cystatin C equation was greater than with other equations, including short MDRD equation [19]. Finally, the comparison of CKD-EPI and MDRD formulas in large cohort of nearly 175,000 primary care patients showed that mean bias, although statistically significant at all age groups, diminished with age, from 13% in the 18–29 years age group to − 7.5% in those aged over 90 years [20]. Authors concluded that, at age over 70 years, there is very little difference between these two equations [20]. Of note, in a subset of very old subjects with measured GFR < 60 ml/min/1.73 m^2^, short MDRD equation was characterized by high specificity, comparable to CKD-EPI_creat_ [17].

To date, patients’ weight demonstrated a strong influence on the discrepancy between C–G and full MDRD formulas in one study [13]. Moreover, short MDRD and other equations’ accuracy were generally lower in patients with diabetic nephropathy [14]. Of importance, an essential aspect of our study is the detailed investigation of demographic and clinical factors, which could markedly influence eGFR values. Most of them, except for age, have not been included in the previous analyses. We would like to emphasize that apart from CKD, the older population is characterized by high comorbidity and large proportion of disabled persons. It is of particular importance that some of these conditions may substantially change the GFR estimation and, in consequence, cause an inappropriate adjustment of medication dosage in such patients. In our study, age, the presence of heart failure, and UACR ≥ 300 mg/g were among the most important factors affecting the relative bias for full MDRD equation, while the occurrence of age and visceral obesity was the factors affecting the relative bias for C–G equation. Of note, multivariable logistic regression analysis confirmed the independent influence of age over 80 years, visceral obesity, and female gender on the C–G equation accuracy, while any for full MDRD equation. The unexpected effect of visceral obesity on the relative bias between C–G and MDRD_short_ equations is probably a consequence of the effect of greater body mass in some very old individuals, resulting in higher calculated values according to C–G formula, that diminish the influence of age, with no effect on eGFR computed with MDRD_short_ formula.

Summarizing the results, we may assume that the interpretation of eGFR values calculated according to the C–G formula should be cautious, especially in the very old female population, as age and gender consist of the main factors causing the substantial bias of C–G formula, with severe sarcopenia as a possible explanation. Moreover, as a similar MDRD and CKD-EPI formulas’ performance was noted within the most clinically relevant GFR range, i.e., below 60 ml/min/1.73 m^2^ [14, 15, 17], with the only exception for formulas based on simultaneous creatinine and cystatin C determination, and keeping in mind the cost-effectiveness issues, the GFR estimation in daily clinical practice may be still reliably calculated using the short MDRD equation. However, in case of GFR estimation for individual drug dosage adjustment, short MDRD formula should not be used interchangeably with C–G formula, and we should keep in mind patient’s comorbidity as an important factor influencing the accuracy of GFR estimation.

The present study has some limitations. As previously mentioned, we were unable to calculate the bias between short MDRD formula and currently recommended by KDIGO CKD-EPI equation, as serum creatinine in the analyzed cohort was not measured by a method calibrated to IDMS. Even more important limitation is related to the lack of clearance-measured GFR. However, this method is not readily applicable in population-based epidemiological studies. Another limitation is caused by the fact that analyzed data were partially obtained via questionnaire. Finally, some selection bias may be related to the proportionally higher rate of exclusion among very old participants who were not able to get into the vertical position needed for measurement of body weight.

Strengths of our study include the detailed characteristics of comorbidities and different aspects of mobility and functional performance, rarely analyzed in other studies. Moreover, using of the Passing–Bablok regression, the least angle square regression as well as the distance-weighted least-squares regression ensure reliable statistical analysis which is in line with the latest guidelines.

## Conclusions

Taking into account that Summaries of Product Characteristics of many medicines still refer to Cockcroft–Gault formula, whereas laboratories worldwide calculate and report the MDRD_short_-based eGFR values, there is a serious inconsistency with possible life-threatening consequences. As our study showed, GFR estimated with MDRD_short_ and Cockcroft–Gault formulas should not be used interchangeably in very old people, especially in females, as well as in dependent patients and subjects with visceral obesity. Thus, the drug dosing adjustment in older patients should not be based on eGFR values routinely reported by local laboratories, but should be calculated according to the reference, i.e., the Cockcroft–Gault equation.
